# Solid-State Structural Transformation in Zn(II) Metal–Organic Frameworks in a Single-Crystal-to-Single-Crystal Fashion

**DOI:** 10.3390/nano13162319

**Published:** 2023-08-12

**Authors:** Jaewook An, Jihye Oh, Uma Kurakula, Dong Hee Lee, Aditya Choudhury, Eunji Lee, Raghavender Medishetty, In-Hyeok Park

**Affiliations:** 1 Graduate School of Analytical Science and Technology (GRAST), Chungnam National University, Daejeon 34134, Republic of Korea; 2 Department of Chemistry, GEC Campus, Indian Institute of Technology Bhilai, Sejbahar, Raipur 492015, India; 3 Department of Chemistry, Gangneung-Wonju National University, Gangneung 25457, Republic of Korea

**Keywords:** metal–organic frameworks, [2 + 2] photoreaction, solid-state structural transformation, single crystal to single crystal, interdigitated framework

## Abstract

Solid-state structural transformation is an interesting methodology used to prepare various metal–organic frameworks (MOFs) that are challenging to prepare in direct synthetic procedures. On the other hand, solid-state [2 + 2] photoreactions are distinctive methodologies used for light-driven solid-state transformations. Meanwhile, most of these photoreactions explored are quantitative in nature, in addition to them being *stereo*-selective and *regio*-specific in manner. In this work, we successfully synthesized two photoreactive novel binuclear Zn(II) MOFs, [Zn_2_(spy)_2_(tdc)_2_] (**1**) and [Zn_2_(spy)_4_(tdc)_2_] (**2**) (where spy = 4-styrylpyridine and tdc = 2,5-thiophenedicarboxylate) with different secondary building units. Both MOFs are interdigitated in nature and are 2D and 1D frameworks, respectively. Both the compounds showed 100% and 50% photoreaction upon UV irradiation, as estimated from the structural analysis for **1** and **2**, respectively. This light-driven transformation resulted in the formation of 3D, [Zn_2_(*rctt*-ppcb)(tdc)_2_] (**3**), and 2D, [Zn_2_(spy)_2_(*rctt*-ppcb)(tdc)_2_] (**4**) (where *rctt* = *regio, cis, trans, trans*; ppcb = 1,3-bis(4′-pyridyl)-2,4-bis(phenyl)cyclobutane), respectively. These solid-state structural transformations were observed as an interesting post-synthetic modification. Overall, we successfully transformed novel lower-dimensional frameworks into higher-dimensional materials using a solid-state [2 + 2] photocycloaddition reaction.

## 1. Introduction

Solid-state structural transformations are interesting reactions in metal–organic frameworks (MOFs) often used to alter the structure of solid-state materials and prepare various exciting materials [1,2,3,4,5,6,7,8,9,10,11,12]. These transformations lead to structural modifications along with changes in the dimensionalities of the frameworks [13,14]. These products are often challenging or almost impossible to obtain in regular synthetic procedures. This change in structure during the structural transformations helps in the modification or fine-tuning of the structure-related properties, which has been significantly established in MOFs over the last three decades [5,15]. Although the developments in crystal engineering in the last two decades paved the way for better design of solid-state materials and MOFs, the synthesis of frameworks that can undergo structural transformation is still a challenge, mainly driven by limited control during MOF syntheses [5,15,16].

Solid-state [2 + 2] photoreactions are one of the prominent photoreactions to prepare highly strained cyclobutane rings in quantitative yields via green synthetic procedures [17,18,19,20]. These solid-state reactions are known to transform molecules in the materials in a *regio*-specific and *stereo*-selective manner, which is difficult to obtain otherwise [21]. Schmidt established the basic criteria in the early 1970s for these solid-state photoreactions [22], and later MacGillivray provided the foundational work in organic materials. Later, Vittal and other groups extended this photoreaction in various materials, including coordination polymers or MOFs for materials with highly strained cyclobutane-based linkers as the post-synthetic modification [18,20,23,24,25,26,27,28,29,30,31]. These transformations also result in the quantitative photoreactions of the linkers in greener processes and quantitative yields. These structural transformations are expected to show that the properties of the materials are different due to having different reactive groups after the photoreaction. These transformations also cause changes in pore size and polarity of the pore surface. For example, before photoreaction, the compound has olefin groups with *sp*^2^ hybrid carbon atoms that interact differently with various guest molecules compared to the cyclobutane rings with *sp*^3^ carbons. Thus, the compounds before and after the photo-transformation have different selectivity and help to tune guest selectivity for various guest molecules [32,33,34]. The major hurdle in structural confirmation of the structural transformation is structural characterization after the photoreaction.

Structural characterization is the major task in most structural transformations, as limited tools are available. In this regard, single-crystal-to-single-crystal (SCSC) structural transformation is one of the unique methods that help elucidate the final structure and mechanism in various reactions/transformations. This SCSC transformation allows for the precise characterization of products at the atomic level unambiguously even after transformation through single-crystal X-ray crystallography (SC-XRD), which is difficult otherwise. However, SCSC transformations are challenging due to the structural change during structural transformation without the loss of single crystallinity. These structural changes in MOFs/CPs often generate strain in the framework and cause the loss of single crystallinity. In a few cases, this also leads to the violent mechanical motions of single crystals, especially those driven by photoreactions [23,35,36,37].

**Figure 1:**
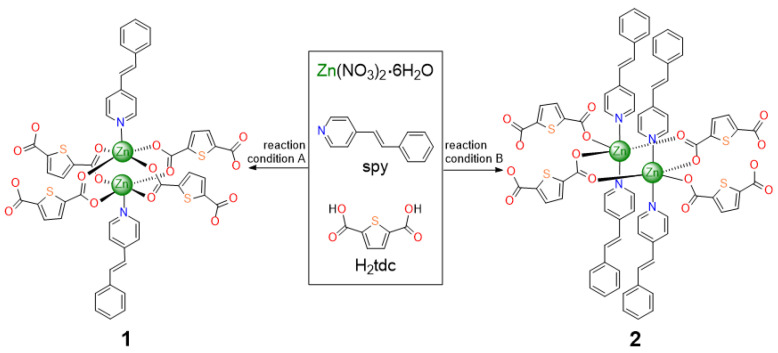
Details of the formation of two Zn(II) metal–organic frameworks (blue: N atom; red: oxygen atom; orange: sulfur atom).

In our current work, we successfully synthesized two novel photoreactive 2D and 1D MOFs for photo-driven structural transformation. These MOFs were synthesized through the systematic control of synthetic procedures (Figure 1). Both compounds are interdigitated in nature and undergo solid-state structural transformation under photo-irradiation, forming higher-dimensional frameworks (2D → 3D; 1D → 2D). This is mainly driven by the head-to-tail [2 + 2] photocycloaddition of spy (spy = 4-styrylpyridine) linkers of these interdigitated structures. This light-driven structural transformation and the final structure of the frameworks were successfully established through SCSC transformation and NMR spectroscopy.

## 2. Materials and Methods

### 2.1. General Procedure

All chemicals were purchased from commercial sources and used as received. All solvents used were of reagent grade. The spy ligand was synthesized via the reported procedure [38,39,40]. The elemental analyses were carried out on a LECO CHNS-932 elemental analyzer. The powder X-ray diffraction (PXRD) patterns were recorded on a Siemens D500 diffractometer with graphite monochromatized Cu-Kα radiation (λ = 1.54056 Å) at room temperature (23 °C). The FT-IR spectra were recorded using a Varian 640-IR FT-IR Spectrometer with KBr pellets. The UV irradiation experiments were carried out on a LUZCHEM UV reactor with an 8 W dark-blue phosphor lamp (300–400 nm) and an Xe lamp at room temperature. The photoreaction was performed by irradiating the sample for 48 h after packing the samples in between the Pyrex glass slides. Raw single crystals were packed between the slides and irradiated under UV light for SCSC transformation or SC-XRD analyses. For the bulk material, the single crystals were ground to a fine powder, packed evenly between the slides, and these slides were flipped at equal intervals of time for uniform photo-irradiation. The photoreaction of these bulk powdered compounds was confirmed through NMR measurements. The NMR measurements were performed using a Bruker AVANCE Neo 400 instrument. These MOFs are not soluble in DMSO-*d*_6_, so a drop of Conc. HNO_3_ was added to dissolve the samples.

### 2.2. Preparation of [Zn_2_(spy)_2_(tdc)_2_] (***1***)

A mixture of spy (12.0 mg, 0.07 mmol), H_2_tdc (tdc = 2,5-thiophenedicarboxylate) (12.1 mg, 0.07 mmol), Zn(NO_3_) · 6H_2_O (20.8 mg, 0.07 mmol) dissolved in DMA (3.0 mL), DMSO (0.5 mL), H_2_O (4.0 mL), and 3 drops of 0.1 *M* NaOH was placed in a 10 mL vial. The vial was kept at 100 °C for 48 h, then cooled to room temperature over 8 h. Yellow plate-shaped crystals were obtained. Anal. Calcd [C_38_H_26_N_2_O_8_S_2_Zn_2_]: C, 54.76; H, 3.14; N, 3.36; S, 7.69. Found: C, 54.54; H, 3.21; N, 3.49; S, 7.30. IR (KBr pellet, cm^−1^): 2911, 2857, 2348, 1615, 1587, 1528, 1413, 1310, 1033, 823, 751, 674, and 619.

### 2.3. Preparation of [Zn_2_(spy)_4_(tdc)_2_] (***2***)

A mixture of spy (25.8 mg, 0.14 mmol), H_2_tdc (12.1 mg, 0.07 mmol), and Zn(NO_3_) · 6H_2_O (20.8 mg, 0.07 mmol) dissolved in DMA (3.0 mL), DMSO (0.5 mL), H_2_O (4.0 mL) and 3 drops of 0.1 *M* NaOH was placed in a 10 mL vial. The vial was kept at 100 °C for 48 h, then cooled to room temperature over 8 h. After 3 days, colorless needle-shaped crystals were obtained. Anal. Calcd [C_64_H_48_N_4_O_8_S_2_Zn_2_]: C, 64.27; H, 4.05; N, 4.68; S, 5.36. Found: C, 64.49; H, 4.12; N, 4.71; S, 5.42. IR (KBr pellet, cm^−1^): 2914, 2859, 2332, 1611, 1582, 1534, 1415, 1323, 1030, 821, 754, 668, and 615.

### 2.4. Preparation of [Zn_2_(rctt-ppcb)(tdc)_2_] (***3***)

Single crystals of **1** were packed between glass slides and then irradiated under UV light for 48 h. Yellow-colored crystals of **3** were obtained. Anal. Calcd [C_38_H_26_N_2_O_8_S_2_Zn_2_]: C, 54.76; H, 3.14; N, 3.36; S, 7.69. Found: C, 54.61; H, 3.19; N, 3.43; S, 7.57. IR (KBr pellet, cm^−1^): 2909, 2853, 2349, 1611, 1583, 1524, 1417, 1316, 1155, 1030, 930, 816, 754, 672, and 620.

### 2.5. [Zn_2_(spy)_2_(rctt-ppcb)(tdc)_2_] (***4***)

Single crystals of **2** were packed between glass slides and then irradiated under UV light for 48 h. Pale yellow-colored crystals of **4** were obtained. Anal. Calcd [C_64_H_48_N_4_O_8_S_2_Zn_2_]: C, 64.27; H, 4.05; N, 4.68; S, 5.36. Found: C, 64.35; H, 3.97; N, 4.88; S, 5.21. IR (KBr pellet, cm^−1^): 2913, 2861, 2338, 1614, 1584, 1531, 1417, 1319, 1156, 1028, 935, 818, 755, 670, and 616.

### 2.6. X-ray Crystallographic Analysis

The crystal structure of the crystallized sample of **1** was determined via single-crystal diffraction methods at the Korea Basic Science Institute (KBSI, Western Seoul Center, Korea). A single crystal of **1** was picked up with paratone oil and mounted on a Bruker D8 Venture PHOTON III M14 diffractometer equipped with a graphite monochromated Mo Ka (λ = 0.71073 Å) radiation source and a nitrogen cold stream (−50 °C). Data collection and integration were performed with SMART APEX3 (Bruker, 2016) and SAINT (Bruker, 2016) [41,42]. The absorption correction was performed via a multi-scan method implemented in SADABS [41]. The structure was solved by direct methods and refined using full-matrix least-squares on *F*^2^ using SHELXTL [43].

The single crystal X-ray diffraction data of **2**–**4** were measured at 223 K with synchrotron radiation (λ = 0.63000 (for **2**), 0.70000 (for **3**), and 0.63000 Å (for **3**)) on a Rayonix MX225HS detector at 2D-SMC with a silicon (111) double-crystal monochromator (DCM) at the Pohang Accelerator Laboratory (PAL), Korea. The PAL BL2D-SMDC program [44] was used for data collection (detector distance: 66 mm, omega scan: Δ*ω* = 3°, exposure time: 1 s/frame), and HKL3000sm (ver. 703r) [45] was used for cell refinement and reduction. The absorption effects were corrected using the multi-scan method (SADABS) [46]. The structures were solved using the direct method (SHELXS) and refined using a full-matrix least-squares technique (SHELXL 2018/3) [47].

In all cases, all nonhydrogen atoms were refined anisotropically, and all hydrogen atoms were placed in idealized positions and refined isotropically in a riding manner along with their respective parent atoms. The relevant crystal data collection and refinement data for the crystal structures are summarized in Table 1.

CCDC 2282494–2282497 contains the supplementary crystallographic data for this paper. These data can be obtained free of charge via http://www.ccdc.cam.ac.uk/conts/retrieving.html (accessed on 18 July 2023) (or from the CCDC, 12 Union Road, Cambridge CB2 1EZ, UK; Fax: +44 1223 336033; E-mail: deposit@ccdc.cam.ac.uk).

## 3. Results and Discussion

### 3.1. Syntheses of MOFs ***1**–**4***

The single crystals [Zn_2_(spy)_2_(tdc)_2_] (**1**) and [Zn_2_(spy)_4_(tdc)_2_] (**2**) were synthesized through differences in the ratio of Zn(NO_3_)_6_ · 6H_2_O, spy, and H_2_tdc, as well as the ratio of mixtures containing DMA, DMSO, 0.1 *M* NaOH, and H_2_O. In the case of MOF **1**, yellow platy crystals were obtained at the solvent–air interface by subjecting the reaction mixture to a solvothermal reaction at 100 °C for 48 h. In the case of MOF **2**, a clear solution was obtained following the completion of the solvothermal reaction. From this clear solution, needle-shaped crystals were obtained after three days. Single-crystal X-ray diffraction (SC-XRD) analysis was utilized for the structural analysis of these compounds. MOFs **3** and **4** were obtained by the UV irradiation of MOFs **1** and **2**, respectively. The photoreaction in single crystals was confirmed through SC-XRD and bulk sample photoreaction was established through ^1^H NMR analysis.

### 3.2. Structural Description of MOF ***1***

**Figure 2:**
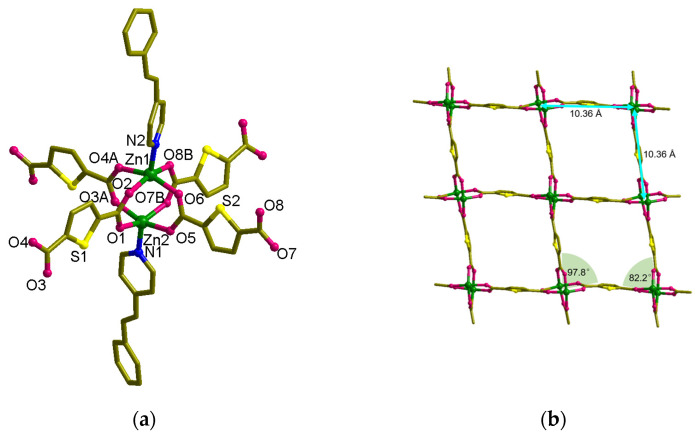

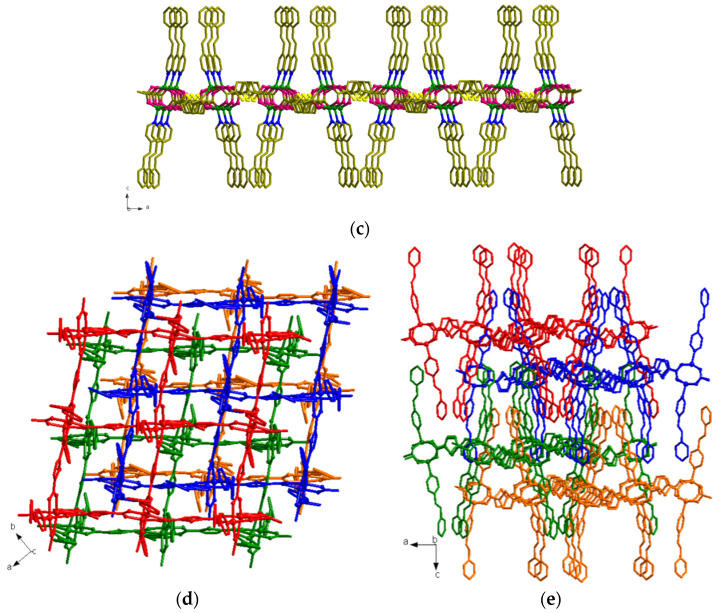
Crystal structure of [Zn_2_(spy)_2_(tdc)_2_] (**1**). (**a**) Coordination environment of Zn(II). (**b**) The (4,4) net formed by Zn_8_(tdc)_4_. (**c**) General view of **1**. (**d**,**e**) Packing structures of **1**. The hydrogen atoms are omitted for clarity.

The solid-state structure of MOF **1**, [Zn_2_(spy)_2_(tdc)_2_], was characterized through single-crystal XRD. This material crystallizes in the *Pbca* space group, and Zn metals are present in the binuclear Zn(II) paddle-wheel secondary building unit (SBU), where both the Zn-atoms are bridged by tdc carboxylate linker (Figure 2). *N*-atoms of spy linker coordinate the apical sites of Zn-paddle wheel SBUs. On the whole, the coordination environment of the Zn(II) atom exhibits a square pyramidal geometry with O atoms at the equatorial and *N*-atoms at the axial sites (Figure 2a). These SBUs are linked by tdc molecules and result in the formation of a 2D framework leading to the formation of a (4,4) layer structure, [Zn_8_(tdc)_4_] (Figure 2b). The width of the (4,4) grid measures 10.36 Å × 10.36 Å (this distance represents the distance between Zn(II) atoms connected by tdc in the grid), and the angles are 82.2° and 97.8°. The total potential solvent area volume in **1** was calculated via PLATON [48]. The unit-cell volume 7371.63 Å^3^ has no residual volume in the lattice.

**Figure 3:**
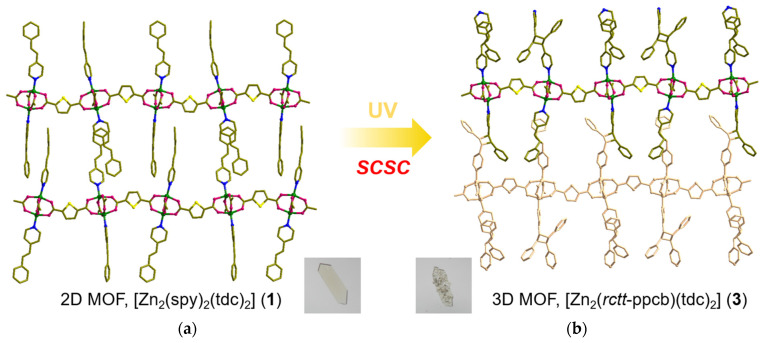
Single-crystal-to-single-crystal transformation (SCSC): the [2 + 2] cycloaddition reaction of (**a**) [Zn_2_(spy)_2_(tdc)_2_] into (**b**) [Zn_2_(*rctt*-ppcb)(tdc)_2_].

The spy ligands are bonded on both sides of the [Zn_8_(tdc)_4_] layers, having head-to-tail (HT) alignment with spy from next layers (Figure 2c,e). This is mainly driven by the π-π interactions between the pyridine and phenyl groups of spy and forms an interdigitated structure (Figure 3a). Although the olefin groups are arranged in a criss-cross arrangement, the distance between the centroids of olefin groups in spy is ca. 3.90 Å, within Schmidt’s criteria. UV irradiation of these compounds showed quantitative photoreaction and formed 3D MOF, [Zn_2_(*rctt*-ppcb)(tdc)_2_] (**3**) (ppcb = 1,3-bis(4′-pyridyl)-2,4-bis(phenyl)cyclobutane), confirmed via SC-XRD (Figure 3b). This quantitative photoreaction was also confirmed through ^1^H NMR spectroscopy (Figure S8 in SI). It can be rationalized that the spy linkers could have undergone pedal motion during or before the photoreaction [49].

### 3.3. Structural Description of MOF ***3***

**Figure 4:**
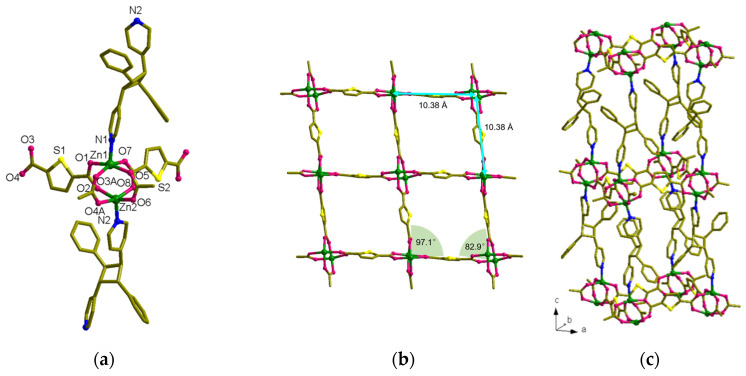

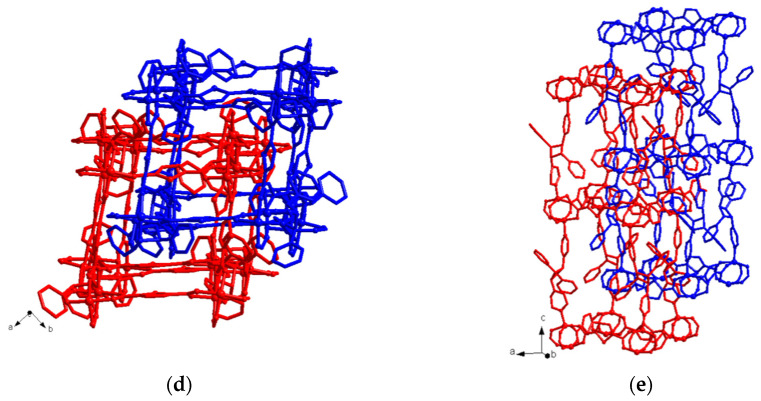
Crystal structure of [Zn_2_(*rctt*-ppcb)(tdc)_2_] (**3**). (**a**) Coordination environments of Zn(II). (**b**) **pcu** repeating unit of **3**. (**c**) The (4,4) net formed by Zn_8_(tdc)_4_. (**d**,**e**) Packing structure of **3**. The hydrogen atoms are omitted.

The structure of the photoproduct, [Zn_2_(*rctt*-ppcb)(tdc)_2_] (**3**) (*rctt* = *regio, cis, trans, trans*; ppcb = 1,3-bis(4′-pyridyl)-2,4-bis(phenyl)cyclobutane) was characterized via SC-XRD (Figure 3b and Figure 4). This compound is crystallized in the *Pbca* space group, the same as its mother material, MOF **1**. Even after photoreaction, the basic framework structure remained the same. The spy linkers underwent photoreaction in a HT manner and formed a stereo-specific photoproduct, *rctt*-ppcb, and formed pillar-layered MOFs where *rctt*-ppcb acts as the pillar between [Zn_8_(tdc)_4_] layers (Figure 4). Meanwhile, the width of the (4,4) grid measures 10.38 Å × 10.38 Å (representing the separation between Zn(II) atoms connected by tdc). The angles were measured to be 89.2° and 96.7°. The spy ligands were arranged head-to-tail at the axial positions of the Zn(II) square pyramidal, while the nitrogen atoms of fully formed *rctt*-ppcb occupied these positions. The distance between Zn-*rctt*-ppcb-Zn···Zn repeating units was 13.93 Å. As a result, the axial spy transforms *rctt*-ppcb, causing the 2D structure to transition into a 3D structure with a **pcu** topology (Figure 3b). This 3D structure exhibits twofold interpenetration. The total potential solvent area volume in **2** was increased to 143.4 Å^3^, [49] which is 2.0% of the unit-cell volume 7108.2 Å^3^ (lower than MOF **1**), confirming the better packing of the framework structure after the structural transformation.

### 3.4. Structural Description of MOF ***2***

**Figure 5:**
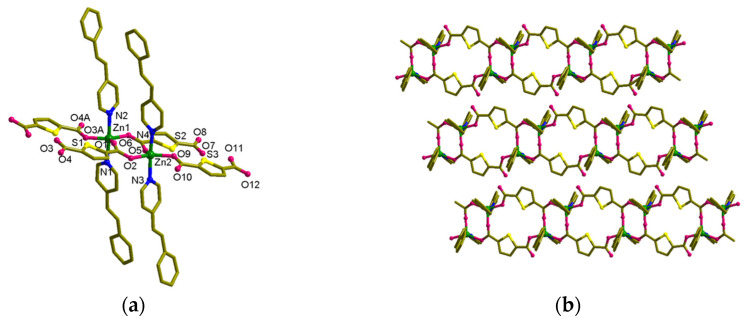

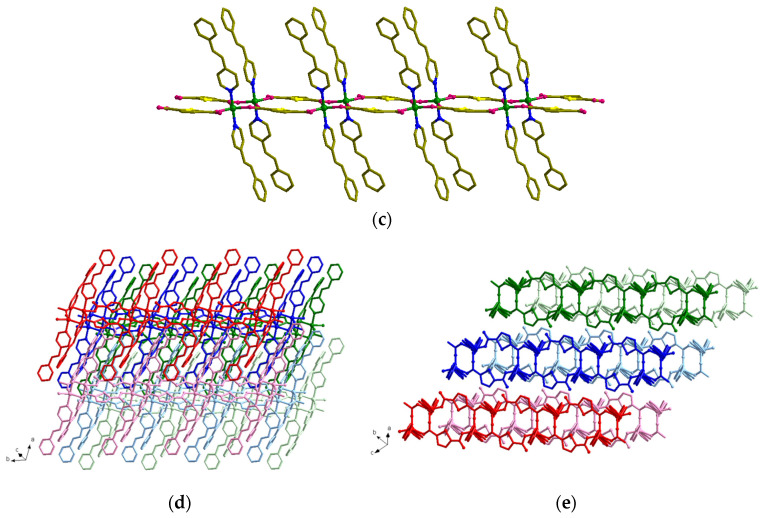
Crystal structure of [Zn_2_(spy)_4_(tdc)_2_] (**2**). (**a**) Coordination environment of Zn(II). (**b**) Top view of **2**. (**c**) General view of **2**. (**d**,**e**) Packing structure of **2**. The hydrogen atoms are omitted.

MOF **2**, [Zn_2_(spy)_4_(tdc)_2_] was obtained as colorless needle-shaped crystals. SC-XRD analysis confirmed that this compound is crystallized in the *P*-1 space group. The Zn(II) metal atoms are present in binuclear repeating units, which are bridged by two carboxylate groups of tdc (Figure 5a). Meanwhile, each Zn(II) atom is also coordinated by tdc carboxylate groups in a monodentate manner and forms a distorted trigonal planar structure. Both the axial sites of Zn(II) are coordinated by spy linkers, resulting in the trigonal bipyramidal coordination geometry of Zn(II) metal atoms, as shown in Figure 5a. Although four tdc linkers coordinate this bimetallic node, which might be due to the bent nature of tdc linkers, this compound resulted in the formation of a 1D framework (Figure 5c). The distance between each node within the repeating unit of [Zn_2_(tdc)_2_] is 8.19 Å. In the repeating unit of [Zn_2_(tdc)_2_], the arrangement of tdc ligands reveals that the sulfur atoms of tdc are arranged in a facing position with the S···S distance being 3.82 Å. Meanwhile, this MOF’s total potential solvent area volume was calculated to be 1.5% (147.2 Å^3^) of the unit-cell volume 9506.1 Å^3^ [49].

**Figure 6:**
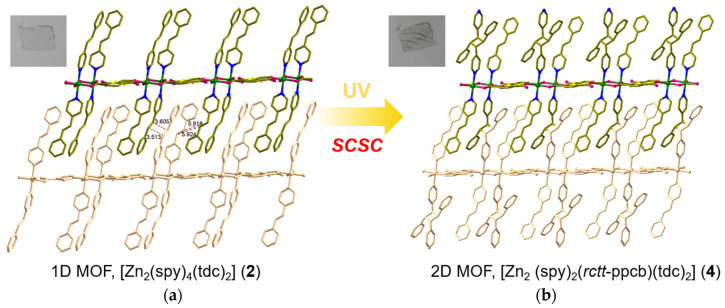
Single-crystal-to-single-crystal transformation (SCSC): the [2 + 2] cycloaddition reaction of (**a**) [Zn_2_(spy)_4_(tdc)_2_] into (**b**) [Zn_2_(spy)_2_(*rctt*-ppcb)(tdc)_2_].

As the distance between Zn(II) atoms in the bridged bimetallic node is 4.24 Å (near Schmidt’s criteria), the spy bound to these Zn(II) atoms are expected to be aligned in a head-to-head arrangement and undergo photoreaction. However, the spy linkers are arranged perpendicularly to each other and the centroids of olefin groups are separated by 4.98 Å, far from Schmidt’s criteria. Fortunately, the spy linkers from the successive layers are aligned in the HT manner, stabilized by strong π-π interactions between the adjacent pyridyl and phenyl groups, and formed an interdigitated structure. However, this alignment is only seen in 50% of the total spy linkers. In these HT-aligned linkers, the centroids of the olefin groups are separated by 3.52 Å and parallelly aligned and ready to react (Figure 6a). Based on this alignment and distance between the olefin groups, photoirradiation of these compounds could show a maximum of 50% photoreaction. As estimated, UV irradiation of this compound showed 50% photoreaction and the final structure of the compound was confirmed through SC-XRD. The photoreaction of the bulk sample was confirmed via ^1^H NMR spectroscopy (Figure S10 in SI).

### 3.5. Structural Description of MOF ***4***

**Figure 7:**
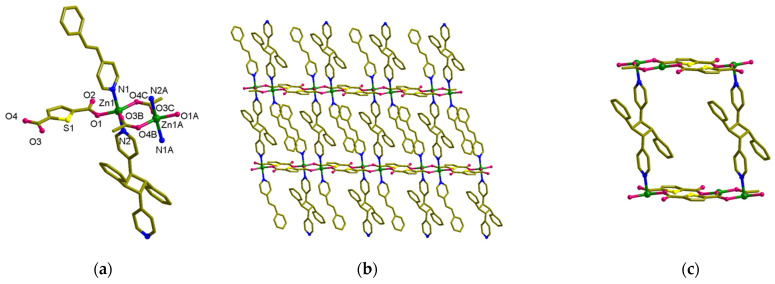

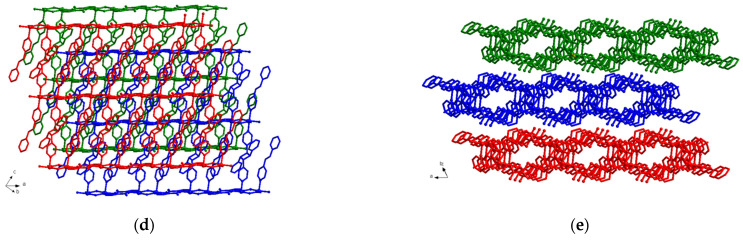
Crystal structure of [Zn_2_(spy)_2_(*rctt*-ppcb)(tdc)_2_] (**4**). (**a**) Coordination environments of Zn. (**b**) Side view of **4**. (**c**) The (4,4) net formed by Zn_4_(*rctt*-ppcb)_2_(tdc)_4_. (**d**,**e**) Packing structure of **4**. The hydrogen atoms are omitted.

Based on the SC-XRD analysis MOF **4**, [Zn_2_(spy)_2_(*rctt*-ppcb)(tdc)_2_] is crystallized in the *P*-1 space group, remaining the same as that of the mother material, MOF **2** (Figure 7). The binuclear SBU of Zn(II) is very similar to **2** (Figure 7a). The distance between the Zn atoms in metal node was slightly increased to 4.27 Å and the distance between Zn atoms (Zn···Zn) is 8.31 Å in the repeating unit of [Zn_2_(tdc)_2_]. During the UV irradiation, only half of the spy ligands underwent the photo-cycloaddition reaction in an HT manner. This resulted in the axial positions of the octahedral Zn(II) center being occupied by one N atom each from the spy and *rctt*-ppcb ligands, with one above and one below. In this case, the *rctt*-ppcb and spy ligands arranged alternatingly, occupying the positions above and below the axis of Zn(II). The distance between Zn-*rctt*-ppcb-Zn···Zn repeating units measures 14.260 Å. Upon undergoing a photochemical reaction, the connectivity of spy ligands in MOF **2** leads to a structural transformation from a 1D arrangement to a 2D configuration, resulting in an increment in the dimensionality of the material. Similarly to the reduction in solvent-accessible space in MOF **3** during the photoreaction, the solvent accessible in this transformation was also reduced to nil, confirming the close packing of the structure during the photoreaction.

## 4. Conclusions

In summary, we demonstrated the solid-state structural transformation of two novel photoreactive interdigitated MOFs. Although both MOFs formed through bimetallic Zn(II) nodes, the structure of these nodes is significantly different due to different experimental conditions. The photoreaction of MOF **1** showed the structural transformation of a 2D interdigitated structure into a doubly interpenetrated 3D pillar layered framework with a **pcu** topology, driven by the quantitative photoreaction of spy linkers. Meanwhile, in the case of MOF **2**, the structural transformation of 1D → 2D MOFs was observed, with only 50% of spy linkers undergoing the photoreaction. The final structure of both these transformations was characterized through SC-XRD due to the successful SCSC transformations. This SCSC transformation confirms a better packing arrangement of molecules that was observed by the reduction in residual solvent compared to its parent MOFs. Overall, this photo-transformation supported us in obtaining MOFs with highly strained cyclobutene-based linkers and high *stereo*-selectivity.

## Figures and Tables

**Table 1:** Crystal and Experimental Data and Refinement Parameters of **1**–**4**.

	1	2	3	4
Formula	C_304_H_208_N_16_O_64_S_16_Zn_16_	C_224_H_168_N_14_O_28_S_7_Zn_7_	C_304_H_208_N_16_O_64_S_16_Zn_16_	C_32_H_24_N_2_O_4_SZn
Formula weight	6667.73	4185.72	6667.73	597.96
Temperature (K)	223(2)	223(2)	223(2)	223(2)
Crystal system	Orthorhombic	Triclinic	Orthorhombic	
Space group	*Pbca*	*P*-1	*Pbca*	*P*-1
*Z*	1	2	1	2
*a* (Å)	15.619(10)	12.327(6)	15.565(3)	10.511(2)
*b* (Å)	13.628(9)	19.995(4)	13.750(3)	12.313(3)
*c* (Å)	34.631(17)	38.859(8)	33.213(7)	12.463(3)
*α* (°)	90	91.48(2)	90	69.62(3)
*β* (°)	90	93.97(4)	90	72.22(3)
*γ* (°)	90	95.486(19)	90	65.06(3)
*V* (Å^3^)	7372(8)	9506(5)	7108(3)	1346.9(6)
*D*_calc_ (g/cm^3^)	1.502	1.462	1.558	1.474
2*θ*_max_ (°)	52.00	52.00	52.00	52.00
*R*_1_, *wR*_2_ [*I* > 2*σ*(*I*)]	0.0618, 0.1721	0.0625, 0.1314	0.0415, 0.1124	0.0346, 0.0966
*R*_1_, *wR*_2_ [all data]	0.0968, 0.2238	0.3039, 0.1549	0.0425, 0.1130	0.0413, 0.0991
Goodness-of-fit on *F*^2^	1.012	1.050	1.081	1.054
No. of reflection used [>2σ(*I*)]	7230 [*R*_int_ = 0.1216]	35774 [*R*_int_ = 0.0743]	9950 [*R*_int_ = 0.0647]	7151 [*R*_int_ = 0.0204]
Refinement	83,844	67,872	72,761	14,241

## Data Availability

The data presented in this study are available in the Supplementary Materials.

## Supplementary Materials

The following supporting information can be downloaded at: https://www.mdpi.com/article/10.3390/nano13162319/s1, Figure S1: Chemical structures used in this work; Figure S2: Synthetic scheme of preparation of **1**–**4**; Figure S3: Alignment of spy ligands in the space of [Zn_8_tdc_4_] net in **1**; Figure S4: Coordination environment of **3** showing the *rctt*-ppcb disordered; Figure S5: PXRD patterns for **1**: (top) as-synthesized and (bottom) simulated from the single crystal X-ray data. The discrepancies in the intensities may be due to preferred orientations of the powder or partial removal of solvents during grinding; Figure S6: PXRD patterns for **2**: (top) as synthesized and (bottom) simulated from the single-crystal X-ray data. The discrepancies in the intensities may be due to preferred orientations of the powder or partial removal of solvents during grinding; Figure S7: ^1^H NMR spectrum of **1**; Figure S8: ^1^H NMR spectrum of 2; Figure S9: ^1^H NMR spectrum of **3**; Figure S10: ^1^H NMR spectrum of **4**. Table S1: Selected bond lengths (Å) and bond Angles (°) for **1**. Table S2: Selected bond lengths (Å) and bond Angles (°) for **2**. Table S3: Selected bond lengths (Å) and bond Angles (°) for **3**. Table S4: Selected bond lengths (Å) and bond Angles (°) for **4**. Table S5: Weighting scheme and the individual weight coefficients for **1**–**4**.
