# Supplementary material for: Solid-State Structural Transformation in Zn(II) Metal–Organic Frameworks in a Single-Crystal-to-Single-Crystal Fashion
Source: Nanomaterials (Basel). 2023 Aug 12;13(16):2319. doi: 10.3390/nano13162319 (PMC10459828; doi:10.3390/nano13162319)
Supplement: Supplementary file 1 [file nanomaterials-13-02319-s001.zip › nanomaterials-2539199-supplementary.pdf]

Supplementary Material

for

Solid-State Structural Transformation in Zn(II) Metal–Organic Frameworks in Single-Crystal-to-Single-Crystal Fashion

Jaewook An [1], Jihye Oh [1], Uma Kurakula [2], Dong Hee Lee [1], Aditya Choudhury[2], Eunji Lee [3], Raghavender Medishetty [2,*] and In-Hyeok Park [1,*]

[1] Graduate School of Analytical Science and Technology (GRAST), Chungnam National University, Daejeon 34134, Republic of Korea

[2] Department of Chemistry, Indian Institute of Technology Bhilai, GEC Campus, Sejbahar, Raipur, Chhattisgarh, India

[3] Department of Chemistry, Gangneung-Wonju National University, Gangneung 25457, Republic of Korea

* Correspondence: raghavender@iitbhilai.ac.in (R.M.) and ipark@cnu.ac.kr (I.-H.P)

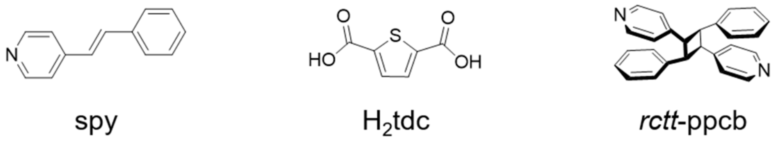

Figure S1. Chemical structures used in this work.

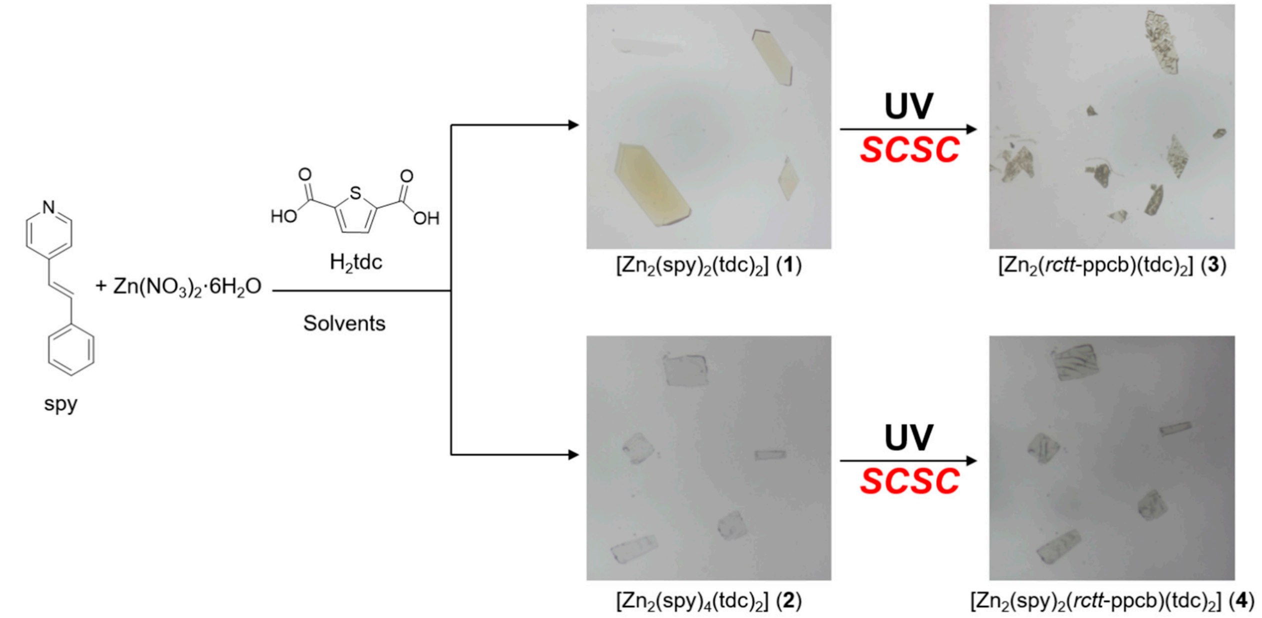

Figure S2. Synthetic scheme of preparation of **1**-**4**.

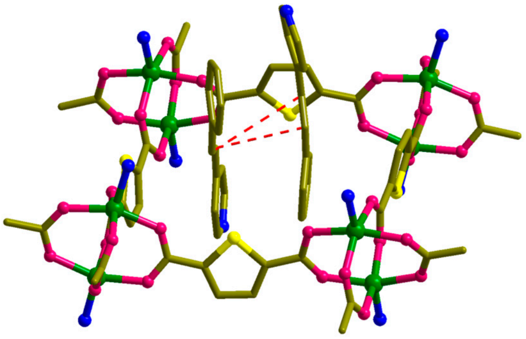

Figure S3. Alignment of spy ligands in the space of [Zn_8tdc_4] net in **1**.

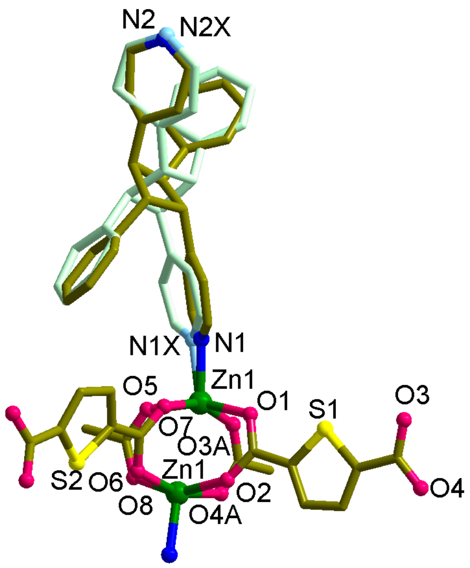

Figure S4. Coordination environment of **3** showing the *rctt*-ppcb disordered.

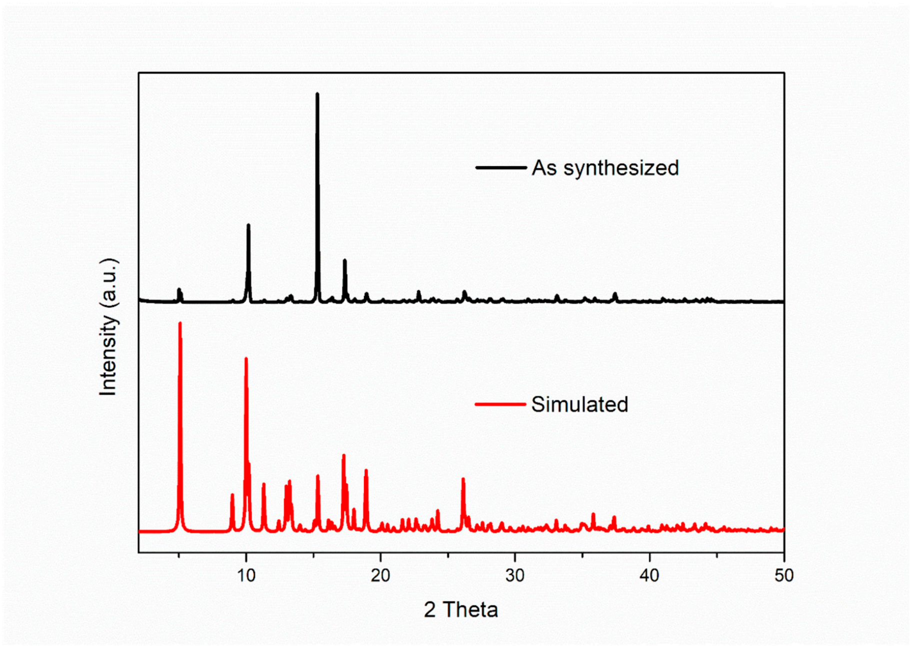

Figure S5. PXRD patterns for **1**: (top) as synthesized and (bottom) simulated from the single crystal X-ray data. The discrepancies in the intensities may be due to preferred orientations of the powder or partial removal of solvents during grinding.

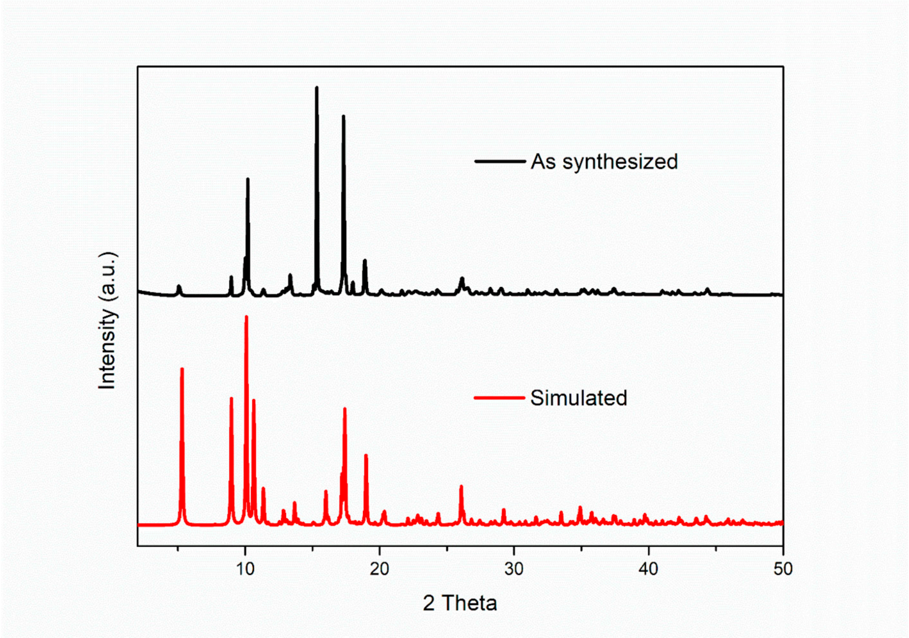

Figure S6. PXRD patterns for **2**: (top) as synthesized and (bottom) simulated from the single crystal X-ray data. The discrepancies in the intensities may be due to preferred orientations of the powder or partial removal of solvents during grinding.

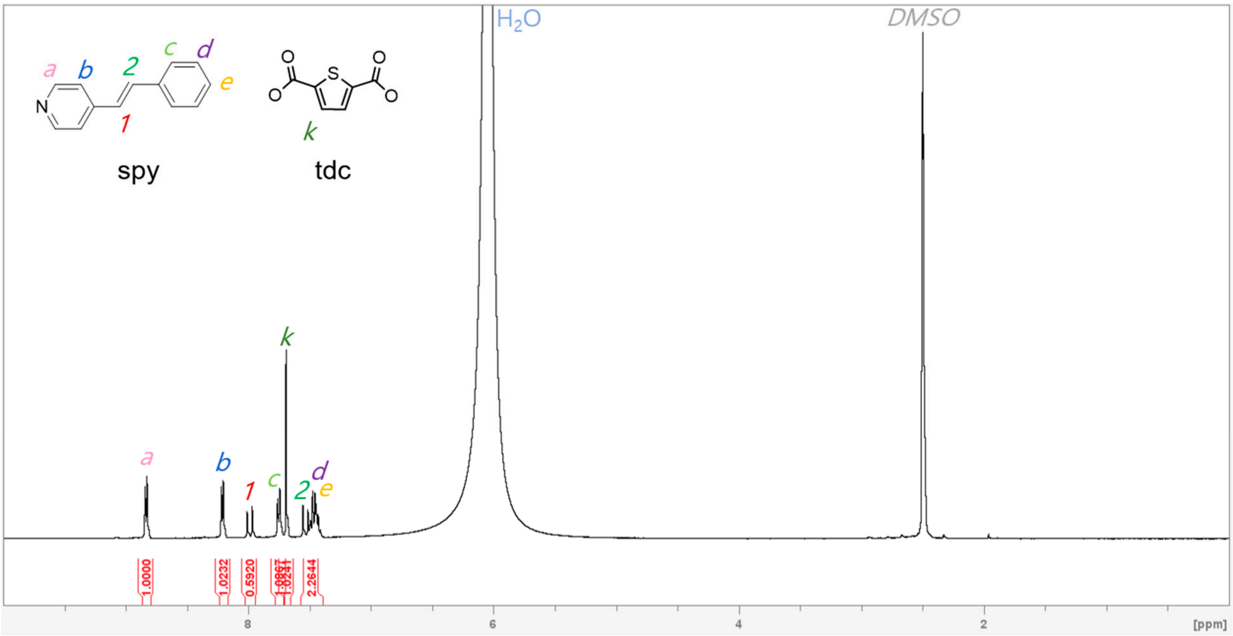

Figure S7. ^{1}H NMR spectrum of **1**.

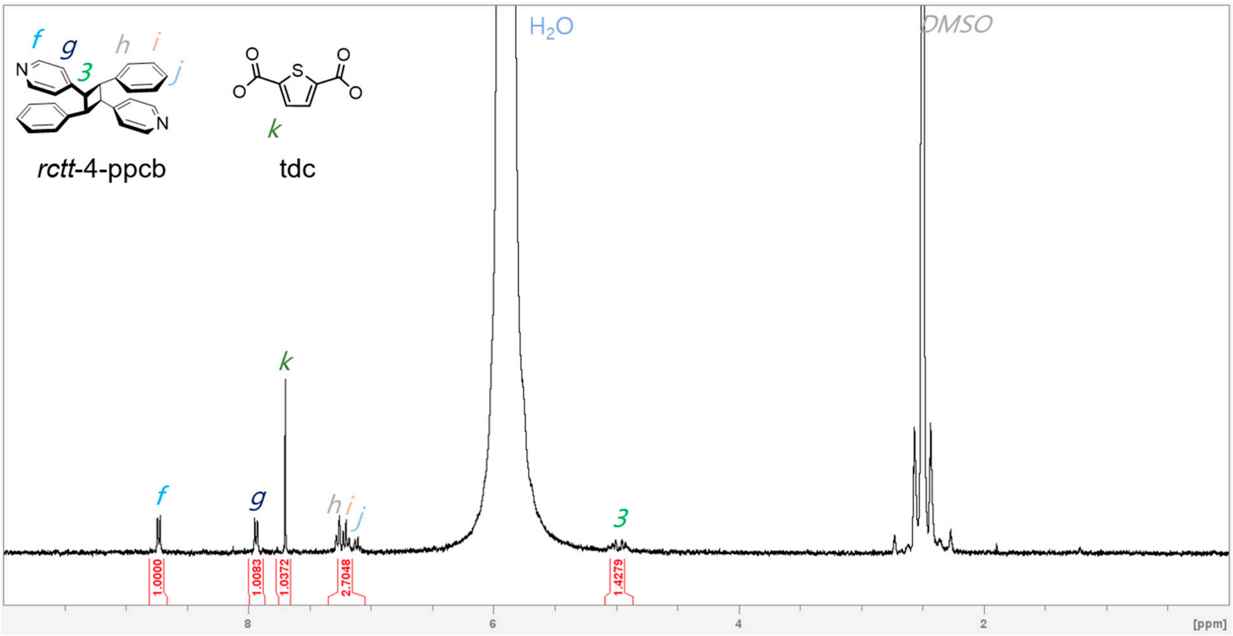

Figure S8. ^{1}H NMR spectrum of **2**.

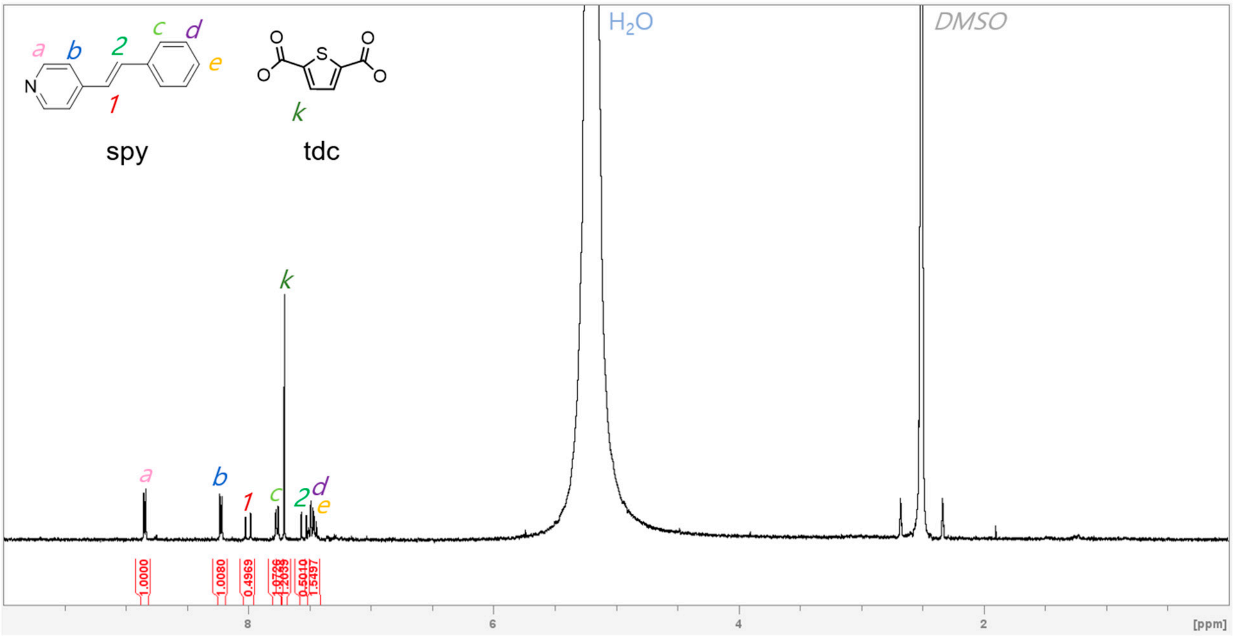

Figure S9. ^{1}H NMR spectrum of **3**.

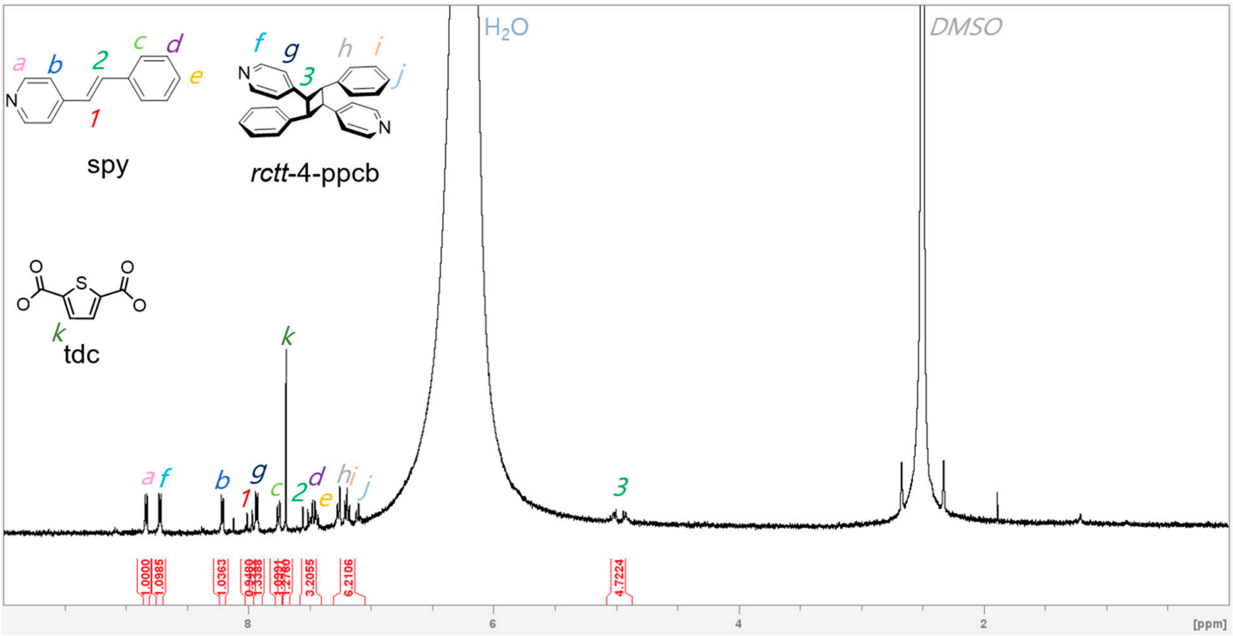

Figure S10. ^{1}H NMR spectrum of **4**.

Table S1. Selected bond lengths (Å) and bond Angles (°) for **1**[a]

Zn1-Zn2	3.0186(16)	Zn1-O2	2.082(4)
Zn1-O4A	2.037(4)	Zn1-O6	2.029(3)
Zn1-O8B	2.060(3)	Zn1-N2	2.039(4)
Zn2-O1	2.058(3)	Zn2-O3A	2.053(3)
Zn2-O5	2.044(3)	Zn2-O7B	2.065(3)
Zn2-N1	2.033(4)	Zn2-Zn1-O2	76.57(10)
Zn2-Zn1-O4A	79.12(11)	Zn2-Zn1-O6	77.90(10)
Zn2-Zn1-O8B	85.96(11)	Zn2-Zn1-N2	173.88(12)
O2-Zn1-O4A	85.69(16)	O2-Zn1- O6	88.41(16)
O2-Zn1-O8B	162.53(15)	O2-Zn1-N2	97.68(15)
O4A-Zn1-O6	157.02(14)	O4A-Zn1-O8B	90.69(15)
O4A-Zn1-N2	102.77(16)	O6-Zn1-O8B	88.32(15)
O6-Zn1-N2	100.02(15)	O8B-Zn1- N2	99.78(16)
Zn1-Zn2-O1	82.12(10)	Zn1-Zn2-O3A	78.80(11)
Zn1-Zn2-O5	80.09(10)	Zn1-Zn2- O7B	71.66(11)
Zn1-Zn2-N1	173.14(11)	O1-Zn2- O3A	88.25(15)
O1-Zn2-O5	87.84(15)	O1-Zn2-O7B	153.60(14)
O1-Zn2-N1	104.69(14)	O3A-Zn2-O5	158.87(14)
O3A-Zn2-O7B	89.64(15)	O3A-Zn2-N1	100.40(16)
O5-Zn2-O7B	84.72(16)	O5-Zn2-N1	100.68(15)
O7B-Zn2-N1	101.57(15)		

[a]Symmetry operations: (A) -x+3/2,y+1/2,z; (B) -x+1/2,y+1/2,z

Table S2. Selected bond lengths (Å) and bond Angles (°) for **2**[a]

Zn1-O1	1.999(11)	Zn1-O3A	1.992(11)
Zn1-O6	2.020(11)	Zn1-N1	2.116(16)
Zn1-N2	2.163(16)	Zn2-O2	2.014(10)
Zn2-O5	2.015(11)	Zn2-O9	1.980(11)
Zn2-N3	2.149(16)	Zn2-N4	2.135(16)
Zn3-O7	1.992(11)	Zn3-O11	2.004(11)
Zn3-O14	2.023(11)	Zn3-N5	2.115(16)
Zn3-N6	2.193(16)	Zn4-O12	2.030(11)
Zn4-O13	2.000(11)	Zn4-O17	1.992(11)
Zn4-N7	2.143(17)	Zn4-N8	2.119(16)
Zn5-O15	2.001(11)	Zn5-O19	1.998(11)
Zn5-O22	2.036(11)	Zn5-N9	2.120(16)
Zn5-N10	2.164(16)	Zn6-O20	2.025(10)
Zn6-O21	1.991(11)	Zn6-O27	2.021(11)
Zn6-N11	2.150(16)	Zn6-N12	2.100(16)
Zn7-O23	1.973(11)	Zn7-O25	2.008(11)
Zn7-O26B	2.025(11)	Zn7-N13	2.124(16)
Zn7-N14	2.189(16)	O1-Zn1-O3A	100.2(5)
O1-Zn1-O6	113.6(5)	O1-Zn1-N1	99.0(5)
O1-Zn1-N2	91.0(5)	O3A-Zn1-O6	146.1(5)
O3A-Zn1-N1	88.8(6)	O3A-Zn1-N2	88.0(5)
O6-Zn1-N1	89.2(5)	O6-Zn1-N2	88.2(5)
N1-Zn1-N2	170.0(5)	O2-Zn2-O5	112.9(4)
O2-Zn2-O9	147.2(4)	O2-Zn2-N3	88.3(5)
O2-Zn2-N4	89.4(5)	O5-Zn2-O9	99.6(5)
O5-Zn2-N4	99.5(5)	O5-Zn2-N3	90.4(5)
O9-Zn2-N3	87.7(5)	O9-Zn2-N4	88.9(5)
N3-Zn2-N4	170.0(5)	O7-Zn3-O11	100.3(4)
O7-Zn3-O14	146.0(4)	O7-Zn3-N5	89.2(5)
O7-Zn3-N6	87.9(5)	O11-Zn3-O14	113.4(5)
O11-Zn3-N5	99.3(5)	O11-Zn3-N6	89.7(5)
O14-Zn3-N5	90.1(5)	O14-Zn3-N6	87.5(5)
N5-Zn3-N6	170.9(5)	O12-Zn4-O13	114.4(5)
O12-Zn4-O17	144.9(4)	O12-Zn4-N7	86.8(5)

O12-Zn4-N8	90.3(5)	O13-Zn4-O17	100.3(5)
O13-Zn4-N7	90.7(5)	O13-Zn4-N8	98.8(5)
O17-Zn4-N7	87.8(5)	O17-Zn4-N8	89.4(5)
N7-Zn4- N8	170.4(5)	O15-Zn5-O19	100.6(5)
O15-Zn5-O22	145.0(5)	O15-Zn5-N9	89.6(6)
O15-Zn5-N10	87.8(5)	O19-Zn5-O22	113.9(5)
O19-Zn5-N9	99.5(5)	O19-Zn5-N10	91.1(5)
O22-Zn5-N9	90.0(5)	O22-Zn5-N10	86.3(5)
N9-Zn5-N10	169.3(5)	O20-Zn6-O21	115.0(5)
O20-Zn6-O27	144.0(4)	O20-Zn6-N11	87.3(5)
O20-Zn6-N12	89.5(5)	O21-Zn6-O27	100.8(5)
O21-Zn6-N12	98.9(5)	O21-Zn6-N11	91.5(5)
O27-Zn6-N11	87.9(5)	O27-Zn6-N12	88.8(5)
N11-Zn6-N12	169.5(5)	O23-Zn7-O25	100.6(4)
O23-Zn7-O26B	145.9(5)	O23-Zn7-N13	89.2(5)
O23-Zn7-N14	87.3(5)	O25-Zn7-O26B	113.1(5)
O25-Zn7-N13	100.1(5)	O26B-Zn7-N13	89.9(5)
O26B-Zn7-N14	87.2(5)	N13-Zn7-N14	169.1(5)

[a]Symmetry operations: (A) -x,-y+3,-z+1; (B) -x+1,-y,-z

Table S3. Selected bond lengths (Å) and bond Angles (°) for **3**[a]

Zn1-Zn2	3.0107(7)	Zn1-O1	2.0311(17)
Zn1-O3A	2.0274(16)	Zn1-O5	2.0438(17)
Zn1-O7B	2.0478(19)	Zn1-N1	2.057(5)
Zn1-N1X	2.040(6)	Zn2-O2	2.0676(16)
Zn2-O4A	2.0362(16)	Zn2-O6	2.0446(16)
Zn2-O8B	2.0544(17)	Zn2-N2C	2.036(6)
Zn2-N2XC	2.035(8)	Zn2-Zn1-O1	89.81(5)
Zn2-Zn1-O3A	78.82(5)	Zn2-Zn1-O5	77.25(5)
Zn2-Zn1-O7B	72.75(6)	Zn2-Zn1-N1	166.24(16)
Zn2-Zn1-N1X	158.68(18)	O1-Zn1-O3A	88.99(8)
O1-Zn1-O5	87.81(8)	O1-Zn1-O7B	162.51(7)
O1-Zn1-N1	96.92(15)	O1-Zn1-N1X	110.61(18)
O3A-Zn1-O5	155.86(7)	O3A-Zn1-O7B	88.96(9)
O3A-Zn1-N1	113.16(17)	O3A-Zn1-N1X	106.65(19)
O5-Zn1-O7B	86.98(9)	O5-Zn1-N1	90.98(16)
O5-Zn1-N1X	96.85(19)	O7B-Zn1-N1	99.85(15)
Zn1-Zn2- O2	68.06(5)	Zn1-Zn2-O4A	79.32(5)
Zn1-Zn2-O6	80.16(4)	Zn1-Zn2-O8B	84.94(5)
Zn1-Zn2-N2C	166.25(17)	Zn1-Zn2-N2XC	167.2(2)
O2-Zn2-O4A	84.01(7)	O2-Zn2-O6	90.56(7)
O2-Zn2-O8B	152.89(7)	O2-Zn2-N2C	100.06(17)
O2-Zn2-N2XC	99.2(2)	O4A-Zn2-O6	159.34(6)
O4A-Zn2-O8B	88.79(7)	O4A-Zn2-N2C	107.09(18)
O6-Zn2-O8B	87.04(7)	O6-Zn2-N2C	93.47(18)
O6-Zn2-N2XC	100.4(2)	O8B-Zn2-N2C	107.03(17)

[a]Symmetry operations: (A) -x+3/2,y+1/2,z; (B) -x+1/2,y+1/2,z; (C) x,-y+1/2,z-1/2

Table S4. Selected bond lengths (Å) and bond Angles (°) for **4**[a]

Zn1-O1	1.9847(13)	Zn1-O3A	2.0169(16)
Zn1-O4B	2.0250(14)	Zn1-N1	2.1672(18)
Zn1-N2	2.1226(17)	O1-Zn1-O3A	97.58(7)
O1-Zn1-O4B	148.17(7)	O1-Zn1-N1	88.44(7)
O1-Zn1-N2	90.14(7)	O3A-Zn1-O4B	113.94(7)
O3A-Zn1-N1	91.27(7)	O3A-Zn1-N2	97.70(7)
O4B-Zn1-N1	86.67(7)	O4B-Zn1-N2	89.88(7)
Zn2-O4X	2.300(10)	Zn2-O4XA	2.300(10)
Zn2-O6	2.306(7)	Zn2-O6A	2.306(7)
N1-Zn1-N2	171.02(6)		

[a]Symmetry operations: (A) -x,-y+1,-z+1; (B) x+1, y, z

Table S5. Weighting scheme and the individual weight coefficients for **1-4**.

**1**	Weighting scheme	calculated
	Weighting details	'w=1/[\s^2^(Fo^2^)+(0.1564P)^2^] where P=(Fo^2^+2Fc^2^)/3'
**2**	Weighting scheme	calculated
	Weighting details	'w=1/[\s^2^(Fo^2^)+(0.0114P)^2^+0.2454P] where P=(Fo^2^+2Fc^2^)/3'
**3**	Weighting scheme	calculated
	Weighting details	'w=1/[\s^2^(Fo^2^)+(0.0468P)^2^+9.5189P] where P=(Fo^2^+2Fc^2^)/3'
**4**	Weighting scheme	calculated
	Weighting details	'w=1/[\s^2^(Fo^2^)+(0.0599P)^2^+0.2107P] where P=(Fo^2^+2Fc^2^)/3'
